# Histopathological and Quantitative Correlates of Intraoperative Narrow Band Imaging in Brain Metastasis Surgery

**DOI:** 10.3390/cancers18193171

**Published:** 2026-10-01

**Authors:** Tadashi Higuchi, Shinsuke Abe, Satsuki Imaoka, Taisei Aoki, Yohei Nounaka, Atsushi Tsukiyama, Fumihiro Matano, Shigeyuki Tahara, Yasuo Murai

**Affiliations:** 1 Department of Neurological Surgery, Nippon Medical School Musashi-Kosugi Hospital, 1-383 Kosugi-machi, Nakahara-ku, Kawasaki 211-8533, Kanagawa, Japantabara@nms.ac.jp (S.T.); 2 Department of Neurological Surgery, Nippon Medical School, 1-1-5 Sendagi, Bunkyo-ku, Tokyo 113-8603, Japan; 3 Department of Neurological Surgery, Nippon Medical School Chiba-Hokusoh Hospital, 1715 Kamagari, Inzai 270-1694, Chiba, Japan

**Keywords:** brain metastasis, narrow-band imaging, exoscope, ImageJ, Fiji, gray value, tumor margin, histopathology, fluorescence-guided surgery

## Abstract

Background/Objectives: Accurate intraoperative identification of the tumor–brain interface is important in brain metastasis surgery, but visual assessment under white light is subjective. Narrow-band imaging (NBI) enhances hemoglobin-dependent optical contrast without an exogenous agent; whether its intraoperative appearance corresponds to tumor tissue, and whether it measurably increases tumor-to-normal image contrast, has not been established in this setting. Methods: In this retrospective, single-center, exploratory study, we evaluated 25 consecutive patients with brain metastases resected using a 4K three-dimensional exoscope equipped with NBI. In three selected patients, spatially adjacent specimens were separately sampled from grayish-appearing and whitish-appearing regions and assessed histopathologically in a non-blinded, descriptive manner. In all 25 patients, paired white-light and NBI images were retrospectively analyzed in Fiji/ImageJ using regions of interest drawn over tissue visually judged to be tumor or normal brain. Results: In the three patients with paired histology, grayish-appearing specimens contained metastatic tumor cells and whitish-appearing specimens did not; this observation derives from a small, selectively sampled subset and should be regarded as preliminary. Across all 25 patients, absolute tumor-to-normal gray-value contrast was significantly greater under NBI than white light (69.628 ± 45.338 vs. 44.287 ± 29.640; mean paired increase, 25.342; 95% CI, 9.169–41.514; *p* = 0.0035; Cohen’s dz = 0.647), although this increase was not observed in all patients. Conclusions: In this exploratory, non-blinded, retrospective study, NBI increased objective image contrast between visually identified tumor and normal brain relative to white light, and its grayish appearance corresponded to tumor tissue in a small histologically sampled subset. These findings support NBI as a feasible, agent-free adjunct that warrants prospective, blinded validation before its diagnostic accuracy or clinical benefit can be assessed.

## Simple Summary

Surgeons removing brain tumors that spread from other cancers need a reliable way to see where the tumor ends and healthy brain begins. We tested narrow-band imaging (NBI), a light-based technique that highlights blood vessels without any drug, in a retrospective, exploratory study of 25 patients having brain metastasis surgery. In three patients, tissue samples suggested that grayish NBI-appearing areas contained tumor cells while adjacent whitish areas did not; this observation was not blinded and is preliminary. Using computer image analysis in all 25 patients, NBI increased visual contrast between tumor and normal brain compared with white light, although this benefit varied and was not seen in every patient. NBI may become a simple, repeatable, drug-free surgical aid, although prospective, blinded studies are needed before it can guide treatment decisions.

## 1. Introduction

Surgery remains an important local treatment for selected patients with symptomatic, large, or diagnostically uncertain brain metastases [1,2]. Although many metastases appear macroscopically well circumscribed, cancer cells can extend into the adjacent brain, and invasive growth patterns have been associated with local recurrence and adverse outcome [3,4,5,6]. The intraoperative definition of a biologically meaningful tumor–brain interface is therefore clinically relevant, particularly when the surgeon must balance local control against preservation of eloquent tissue.

Fluorescence-guided surgery with 5-aminolevulinic acid (5-ALA) improves complete resection of contrast-enhancing malignant glioma and is supported by randomized evidence in that setting [7,8]. Its performance in brain metastases is less consistent: reported fluorescence rates vary substantially across and within histological subtypes, and cavity fluorescence may lack specificity for residual metastasis [9,10,11,12]. These limitations motivate complementary visualization strategies with different contrast mechanisms.

Narrow-band imaging uses restricted blue and green illumination bands centered near hemoglobin absorption peaks (approximately 415 and 540 nm), thereby emphasizing superficial and subsurface microvascular architecture and small color differences [13,14]. NBI is established in gastrointestinal and urological endoscopy and has improved optical detection and delineation of epithelial neoplasia [15,16,17]. Neurosurgical experience remains limited, although NBI-guided neuroendoscopic biopsy has demonstrated that the technique can enhance abnormal intraventricular vascular patterns [18]. A recent illustrative case has also reported that NBI can help identify residual vascular abnormality during intracranial vascular surgery, suggesting a broader potential role for intraoperative NBI beyond neuroendoscopy [19]. To our knowledge, the present study is the first to combine histopathological comparison with quantitative image-contrast analysis of NBI specifically in brain metastasis surgery.

Modern 4K three-dimensional exoscopes permit rapid switching among white-light, fluorescence, angiographic, and NBI modes while maintaining an ergonomic heads-up view [20,21,22,23]. NBI requires no drug administration and can be repeated throughout surgery; however, hemoglobin absorption also makes the method vulnerable to blood in the operative field. Moreover, visual impressions require objective validation before NBI can be considered more than a subjective adjunct.

This study was designed around two complementary validation axes: (1) the spatial correspondence of grayish-appearing and whitish-appearing regions with histopathological tumor presence or absence, and (2) objective quantification of the tumor-to-brain contrast in paired white-light and NBI images using Fiji/ImageJ [24,25]. We hypothesized that grayish-appearing regions under NBI would correspond to metastatic tumor and that NBI would increase the gray-value contrast between tumor and adjacent brain parenchyma relative to white-light imaging. The primary objective of this study was to determine, in a retrospective paired-image analysis, whether NBI increases quantitative gray-value contrast between visually identified tumor and adjacent normal brain relative to white light. The histopathological comparison in three selected patients was a secondary, exploratory observation intended to provide preliminary biological plausibility for the visual NBI appearance, and was not designed or powered to establish diagnostic accuracy for tumor identification or margin delineation.

## 2. Materials and Methods

### 2.1. Patient Population

This single-center retrospective observational study included 25 consecutive patients with histopathologically confirmed brain metastasis who underwent resection using an NBI-capable exoscopic system at Nippon Medical School Hospital from June 2022 through June 2025. During this period, approximately 35 patients underwent surgical resection of brain metastasis at this center; ten were excluded because dedicated NBI was not performed or recorded during their operation, or to avoid counting the same patient more than once, yielding the final non-duplicated cohort of 25 patients (Supplementary Figure S1). All 25 patients had complete paired white-light and NBI images suitable for quantitative analysis. Inclusion criteria were: age ≥ 18 years; intra-axial brain metastasis; availability of interpretable NBI recordings; and availability of paired white-light and NBI frames depicting adjacent tumor and brain tissue. Patients with primary glioma, primary central nervous system lymphoma, non-diagnostic histology, inadequate image quality, or loss of correspondence between white-light and NBI frames were excluded from the quantitative analysis. In all 25 patients, the brain metastasis represented disease recurrence or progression despite prior systemic chemotherapy directed at the primary tumor. Tumor volume and the presence of intratumoral hemorrhage or necrosis were not systematically measured in this study and are therefore not reported in Table 1.

The protocol was approved by the Ethics Committee of Nippon Medical School (approval No. R1-11-1212), originally granted on 30 March 2020 (valid through 31 March 2025) and subsequently renewed on 24 February 2024, extending validity through to 31 March 2033; the original approval therefore predates and continuously covers the entire study period. Patient consent was obtained through an opt-out process. The study was conducted in accordance with the Declaration of Helsinki.

### 2.2. Surgical Procedure and ORBEYE/NBI

Operations were performed using a 4K three-dimensional exoscopic system (ORBEYE^®^, Olympus, Tokyo, Japan). Resection proceeded primarily under white light. After exposure of the tumor–brain interface and adequate hemostasis, the operator switched to NBI using the camera control. No exogenous contrast agent was required for NBI. White-light and NBI views were recorded from the same operative field whenever feasible, with the exoscope position, zoom, focus, viewing angle, and field composition maintained as closely as possible. All operations, and all intraoperative grayish-appearing/whitish-appearing assessments, were performed by the same single surgeon (the corresponding author), which provided consistency of image acquisition and visual interpretation across the cohort but precluded assessment of inter-observer variability. Exposure, gain, and white balance were adjusted automatically by the exoscope system when switching between white-light and NBI modes and were not held to fixed manual settings.

**Figure 1:**
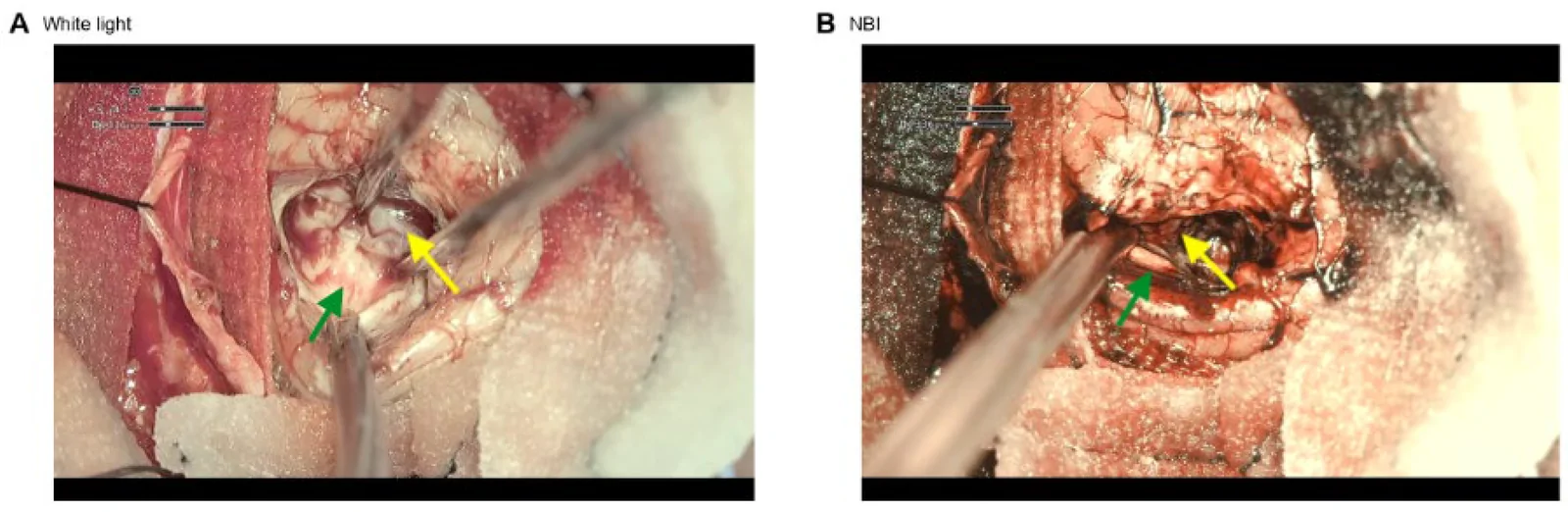
Representative intraoperative appearance under white light and NBI. (**A**) Under white-light illumination, the distinction between tumor and adjacent brain parenchyma is subtle; the yellow arrow indicates the tumor-associated region and the green arrow indicates adjacent normal brain parenchyma. (**B**) Under NBI, the same tumor-facing region (yellow arrow) shows a grayish appearance that contrasts with the surrounding whitish brain tissue (green arrow).

Under NBI, tumor-facing tissue typically appeared grayish or brown-gray relative to adjacent white matter, which retained a whitish appearance resembling normal brain parenchyma (Figure 1). These descriptive categories reflect the operating surgeon’s visual impression under NBI and are not diagnostic classifications; we therefore refer to grayish-appearing and whitish-appearing regions rather than “NBI-positive” and “NBI-negative” elsewhere in this manuscript. Blood-contaminated fields were irrigated and hemostasis was obtained before interpretation because blood caused marked darkening and loss of discrimination. Use of 5-ALA/BL400 or indocyanine green/IR800 for clinical purposes was recorded but did not define NBI appearance.

### 2.3. Tissue Sampling

When anatomically and functionally safe, separate specimens were obtained from a grayish-appearing region and an adjacent whitish-appearing region in the same operative field. Patients selected for this paired sampling were those in whom both sites could be biopsied safely with respect to eloquent versus non-eloquent cortex, rather than those chosen because their NBI appearance was judged to be particularly distinct. Sampling locations were documented on still frames or video captures immediately before removal. Specimens were handled separately and labeled according to intraoperative location. Paired sampling was performed in three selected patients, yielding three grayish-appearing and three whitish-appearing specimens. Sampling of apparently normal tissue was not performed when it would increase neurological risk. The exact anatomical distance between paired sampling sites was not prospectively recorded.

### 2.4. Histopathology

**Figure 2:**
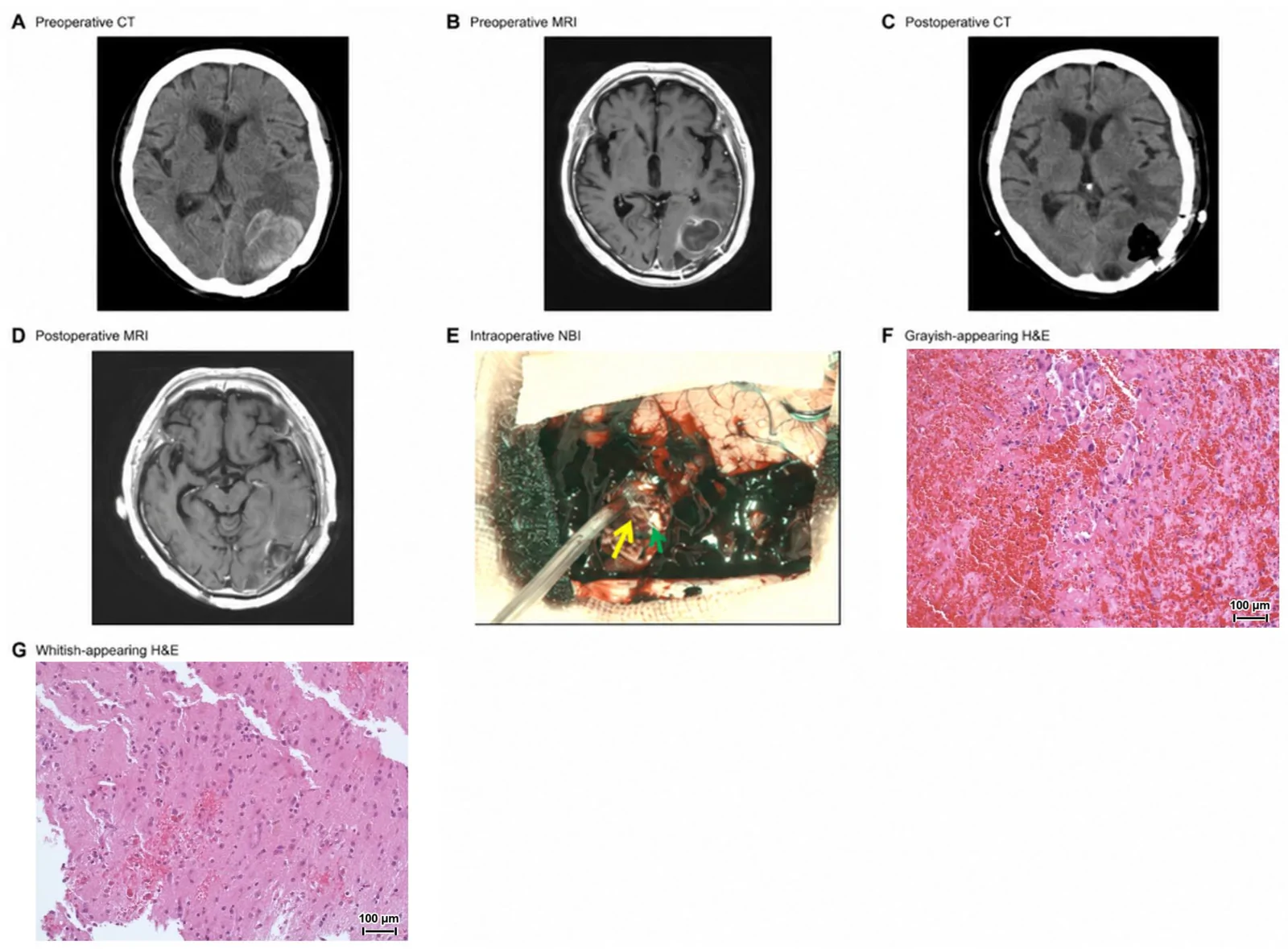
Patient No. 14: brain metastasis from anaplastic thyroid carcinoma. (**A**,**B**) Preoperative CT and MRI. (**C**,**D**) Postoperative CT and MRI. (**E**) Intraoperative NBI appearance; the yellow arrow indicates the tumor-associated grayish-appearing region, and the green arrow indicates the whitish-appearing adjacent white matter. (**F**,**G**) Paired H&E-stained sections (original magnification ×20): tumor cells are present in the grayish-appearing region (**F**) but absent in the whitish-appearing region (**G**).

**Figure 3:**
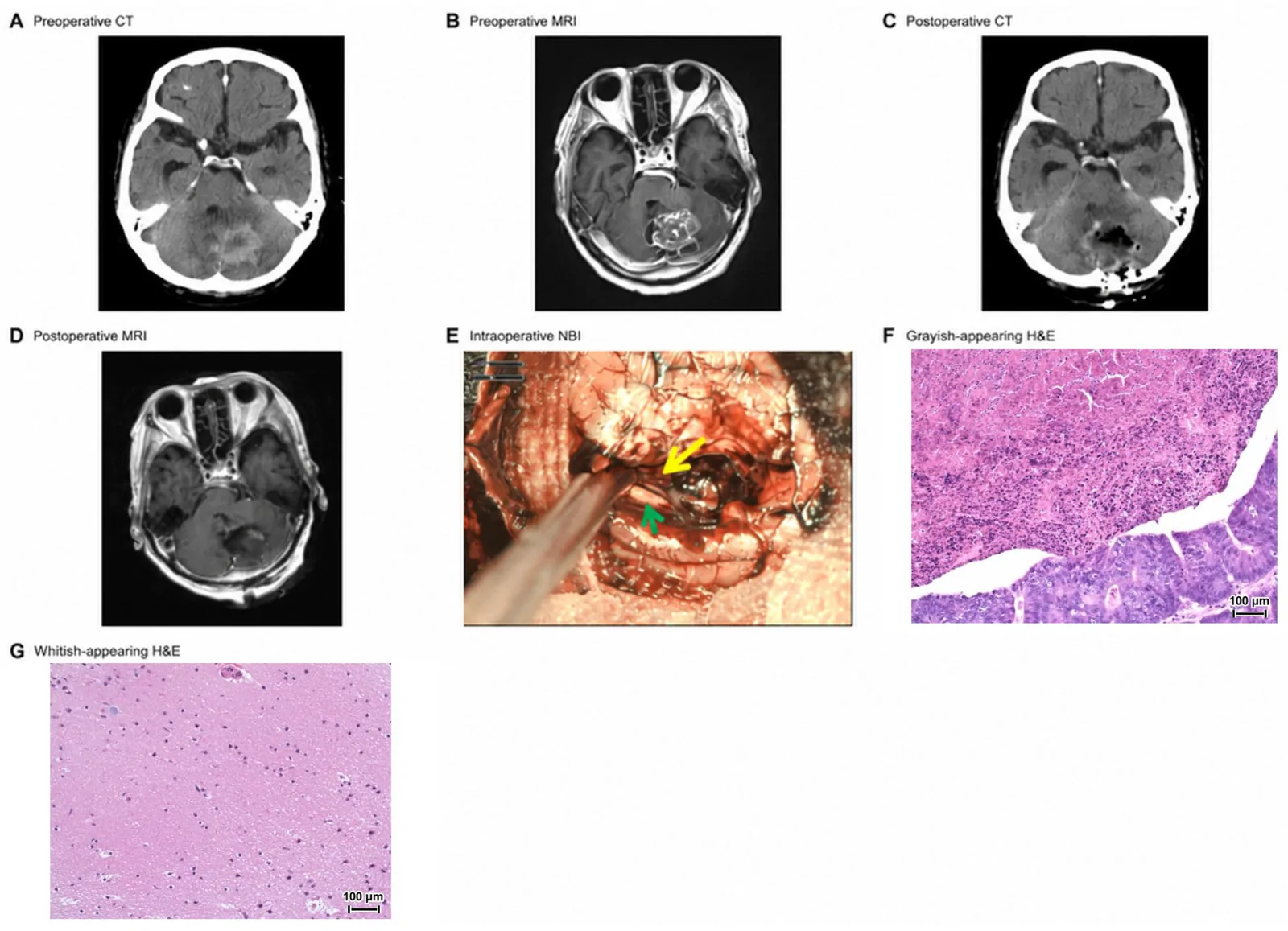
Patient No. 19: brain metastasis from colorectal adenocarcinoma. (**A**,**B**) Preoperative CT and MRI. (**C**,**D**) Postoperative CT and MRI. (**E**) Intraoperative NBI appearance; the yellow arrow indicates the tumor-associated grayish-appearing region, and the green arrow indicates the whitish-appearing adjacent white matter. (**F**,**G**) Paired H&E-stained sections (original magnification ×20): tumor cells with nuclear atypia are present in the grayish-appearing specimen (**F**) but absent in the whitish-appearing specimen (**G**).

**Figure 4:**
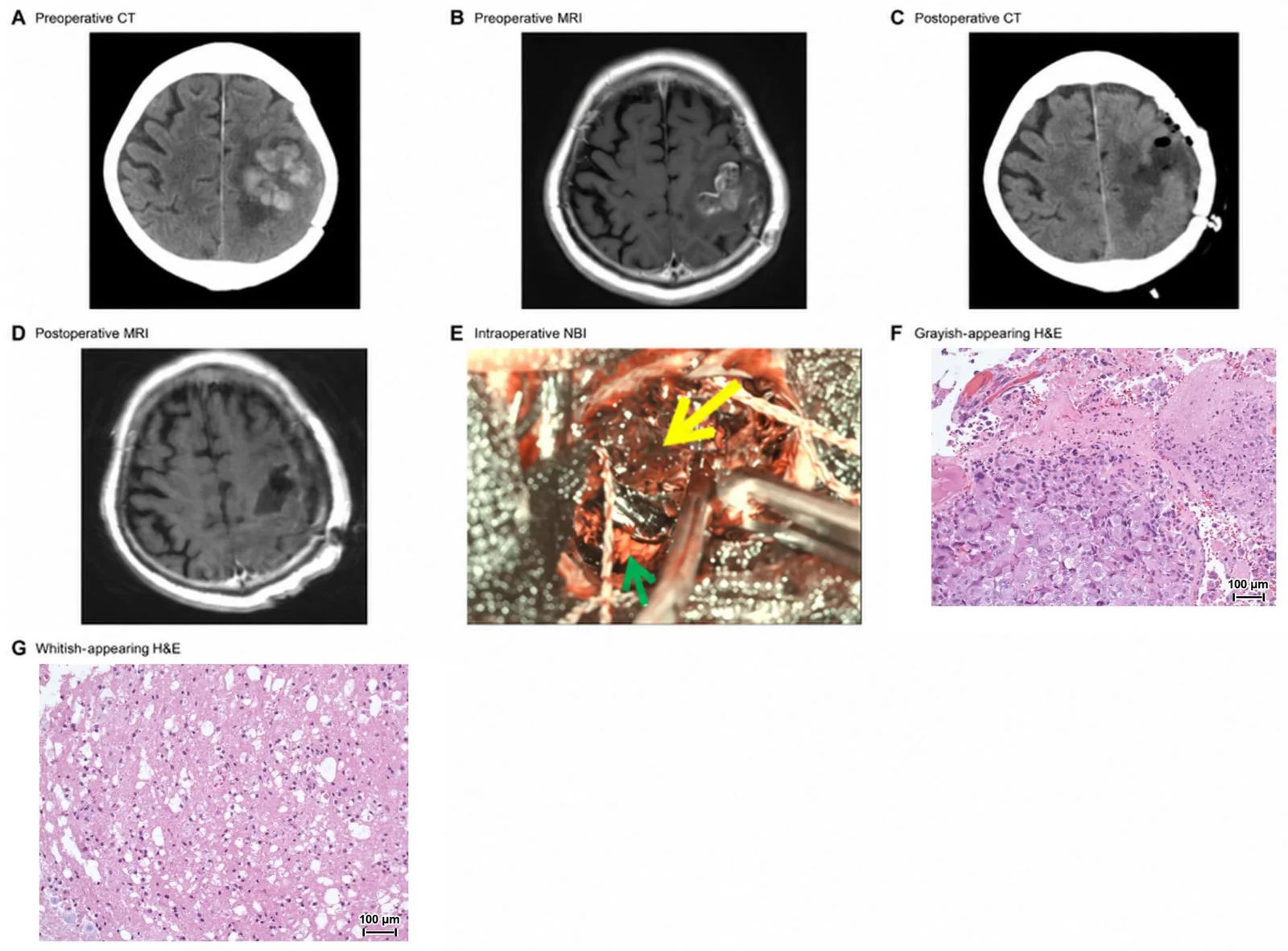
Patient No. 20: brain metastasis from lung adenocarcinoma. (**A**,**B**) Preoperative CT and MRI. (**C**,**D**) Postoperative CT and MRI. (**E**) Intraoperative NBI appearance; the yellow arrow indicates the tumor-associated grayish-appearing region, and the green arrow indicates the whitish-appearing adjacent white matter. (**F**,**G**) Paired H&E-stained sections (original magnification ×20): tumor cells are present in the grayish-appearing specimen (**F**) but absent in the whitish-appearing specimen (**G**).

Specimens were fixed in formalin, embedded in paraffin, sectioned, and stained with hematoxylin and eosin. The grayish-appearing and whitish-appearing specimens were submitted as separate samples, and a pathology report documenting the presence or absence of tumor was returned for each sample. The histopathological images shown in Figure 2, Figure 3 and Figure 4 were obtained from the submitted samples. Experienced neuropathologists assessed metastatic tumor cells, nuclear atypia, cellularity, and infiltration into brain parenchyma. The primary histopathological endpoint was binary tumor presence versus absence in each spatially registered specimen. Neuropathologists were not blinded to the intraoperative NBI classification: each specimen was submitted to pathology already labeled according to its intraoperative grayish-appearing or whitish-appearing designation, consistent with the descriptive, non-blinded nature of this exploratory histopathological comparison.

### 2.5. Quantitative Image Analysis

Digital still frames were exported from recorded surgeries and analyzed in Fiji/ImageJ (version 1.54p; National Institutes of Health, Bethesda, MD, USA) [24,25]. For each eligible field, a white-light frame and an NBI frame depicting the same tumor–brain interface were selected. Frames with major camera movement, defocus, saturation, obscuring instruments, or visible blood over either ROI were excluded; the exact number of frames excluded specifically for blood contamination was not separately logged, but only fields judged free of contamination sufficient to compromise measurement were analyzed. Image selection and measurement were performed jointly by two neurosurgeons: the corresponding author and one assistant professor. Formal blinding of the image analysts to the histopathological results was not documented.

### 2.6. ROI Definition and Gray-Value/Contrast Metrics

**Figure 5:**
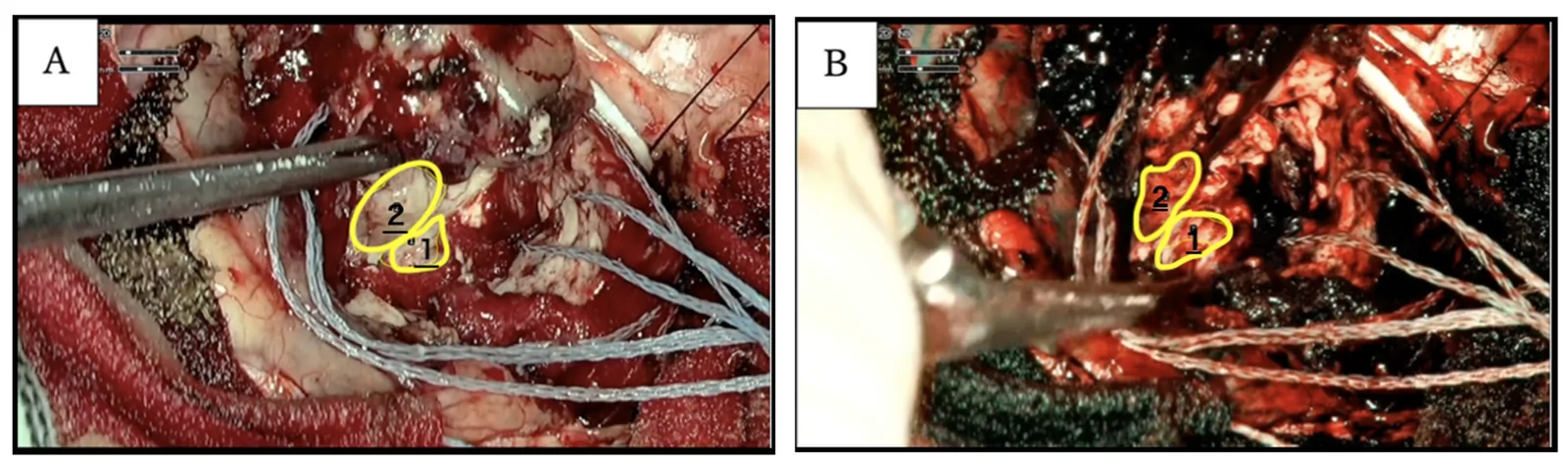
Example of ROI placement for gray-value measurement. (**A**) White-light image and (**B**) corresponding NBI image from the same operative field. In both panels, the yellow outline demarcates the boundary of each freehand region of interest (ROI); ROI 1 represents adjacent normal brain tissue and ROI 2 represents tumor. Freehand ROIs with a maximum diameter of 5 mm were placed at corresponding anatomical locations.

Within each paired image, one freehand ROI was placed over tissue judged to represent tumor and a second freehand ROI over adjacent normal brain parenchyma (Figure 5). Each ROI was defined with a maximum diameter of 5 mm. Corresponding tumor and normal-tissue sites were identified in the paired white-light and NBI frames from the same operative field using visible anatomical landmarks. The paired ROIs were positioned in spatially close areas and matched as closely as possible for location and area to reduce illumination-gradient effects.

Images were converted to 8-bit grayscale using Fiji/ImageJ’s standard RGB-to-grayscale conversion (Image > Type > 8-bit), without selective use of any individual red, green, or blue channel, yielding pixel intensities from 0 (black) to 255 (white). Mean gray value was measured for each ROI. The primary contrast metric was the absolute gray-value difference: ΔGV = |GVtumor − GVnormal|. A secondary normalized contrast metric was calculated as Cnorm = |GVtumor − GVnormal|/(GVtumor + GVnormal). All measurements were retained at the patient/image-pair level. In the 22 patients without paired histological sampling, tumor and normal-tissue ROIs were selected based on the surgeon’s visual impression of the operative field (informed by preoperative imaging) rather than independent histological confirmation; consequently, the quantitative contrast comparison in this study measures whether NBI increases optical contrast between regions visually believed to be tumor and normal brain, and does not by itself constitute an independent test of NBI’s ability to correctly identify tumor tissue.

### 2.7. Statistical Analysis

Continuous variables were summarized as mean ± standard deviation or median with interquartile range, as appropriate. Because white-light and NBI measurements were obtained from the same patient and operative field, all comparisons were paired. Normality of paired differences was evaluated using the Shapiro–Wilk test. As paired differences did not significantly depart from normality, paired-samples t tests were used for the primary analyses. Results are reported as mean paired differences, 95% confidence intervals (CIs), exact two-sided p values, and Cohen’s dz, calculated as the mean paired difference divided by the standard deviation of the paired differences. Wilcoxon signed-rank tests were performed as sensitivity analyses.

For histopathological concordance, findings from the three selected patients with spatially registered paired grayish-appearing and whitish-appearing samples were summarized descriptively. Sensitivity, specificity, and predictive values were not calculated because paired margin sampling was selective and constrained by neurological safety. Analyses were performed using R version 4.5.2. A two-sided *p* < 0.05 was considered statistically significant. No missing values were imputed. No multiplicity adjustment was applied because the comparison of absolute tumor-to-normal contrast between NBI and white light was prespecified as the primary quantitative analysis. No a priori sample-size or power calculation was performed; all consecutive patients meeting the inclusion criteria during the study period were analyzed, consistent with the exploratory nature of this study.

## 3. Results

### 3.1. Patient and Lesion Characteristics

All 25 included patients had complete clinicopathological and gray-value data. The mean age was 68.5 ± 9.4 years, and the median age was 68 years (range, 50–82 years). Seventeen patients (68.0%) were male and eight (32.0%) were female. Primary sites were colon (*n* = 7), lung (*n* = 5), thyroid (*n* = 3), skin (*n* = 2), bladder (*n* = 2), esophagus (*n* = 2), uterus (*n* = 1), liver (*n* = 1), ureter (*n* = 1), and breast (*n* = 1). The clinicopathological characteristics are summarized in Table 1.

### 3.2. Intraoperative NBI Appearance

After hemostasis, metastatic tumor or the tumor-facing margin commonly appeared grayish under NBI, whereas adjacent white matter appeared whitish. This distinction was observed across metastases from multiple primary sites. Active bleeding or residual blood produced diffuse darkening and reduced interpretability. Paired images suitable for quantitative analysis were available in all 25 included patients.

### 3.3. Histopathological Correlation

Spatially registered paired tissue sampling was available in three selected patients, yielding three grayish-appearing and three whitish-appearing specimens. In the representative intraoperative images (Figure 2, Figure 3 and Figure 4), yellow arrows indicate tumor-associated grayish-appearing regions and green arrows indicate whitish-appearing adjacent white matter. In each patient, the grayish-appearing specimen contained metastatic tumor cells with nuclear atypia, whereas the spatially adjacent whitish-appearing specimen showed brain parenchyma without identifiable tumor cells. Because sampling was selective and safety-constrained, these observations were treated as preliminary, hypothesis-generating histopathological observations rather than diagnostic validation, and were not used to calculate diagnostic sensitivity or specificity.

Case 1 was a 77-year-old man with anaplastic thyroid carcinoma metastatic to the left occipital lobe (Figure 2); Case 2 was a 72-year-old man with colorectal adenocarcinoma metastatic to the left cerebellar hemisphere (Figure 3); and Case 3 was a 74-year-old man with lung adenocarcinoma metastatic to the left frontal lobe (Figure 4).

### 3.4. Quantitative Gray-Value and Contrast Analysis

**Figure 6:**
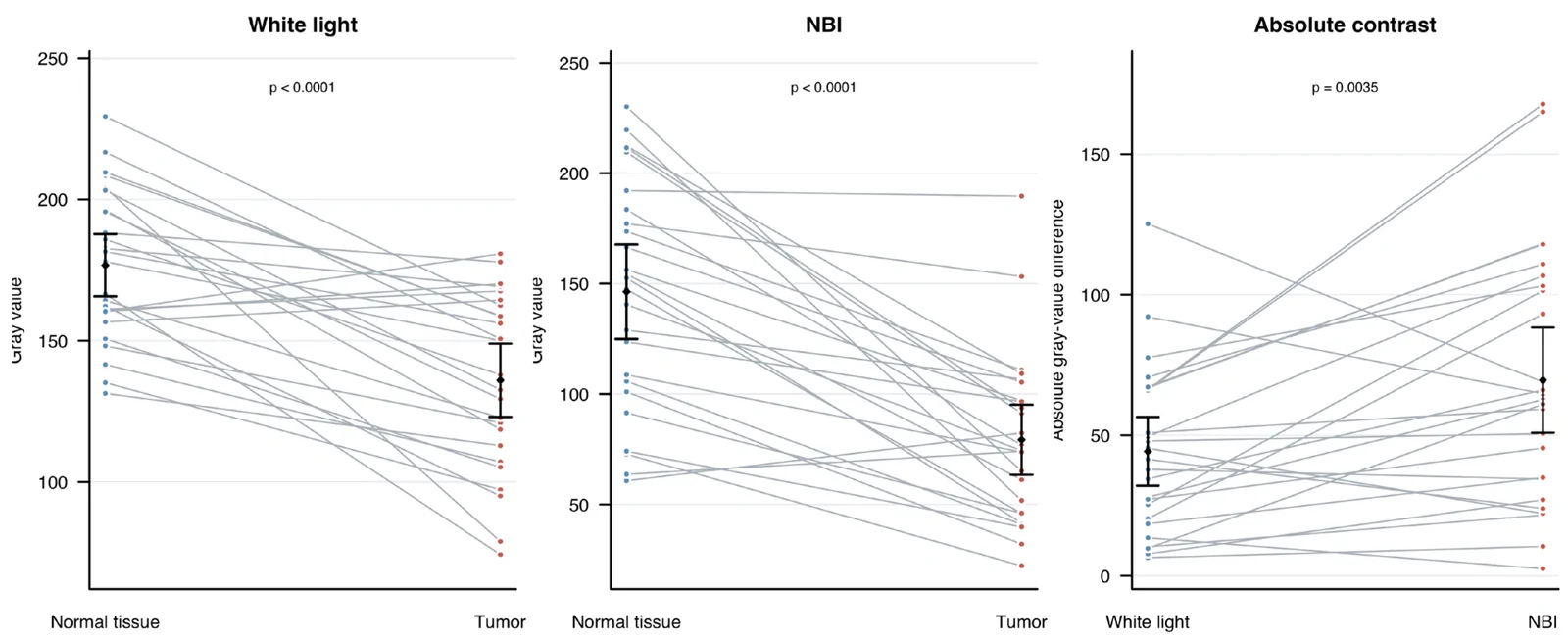
Quantitative comparison of white-light and NBI gray values. Paired patient-level observations are shown for normal tissue versus tumor under white light (**left**), normal tissue versus tumor under NBI (**center**), and absolute tumor-to-normal contrast under white light versus NBI (**right**). Black diamonds and error bars indicate means and 95% CIs. All comparisons used paired-samples t tests (*n* = 25).

Quantitative analysis included all 25 patients and 25 paired white-light/NBI fields (Figure 6). Under white light, mean gray values were 176.731 ± 26.689 for normal tissue and 135.992 ± 31.430 for tumor tissue. The mean paired difference was 40.739 gray-value units (95% CI, 26.483–54.995; t(24) = 5.898; *p* < 0.0001; Cohen’s dz = 1.180). Under NBI, mean gray values were 146.326 ± 51.877 for normal tissue and 79.266 ± 38.443 for tumor tissue. The mean paired difference was 67.060 units (95% CI, 46.749–87.371; t(24) = 6.814; *p* < 0.0001; Cohen’s dz = 1.363).

Absolute tumor-to-normal contrast was 44.287 ± 29.640 under white light and 69.628 ± 45.338 under NBI. NBI increased absolute contrast by a mean of 25.342 units (95% CI, 9.169–41.514; t(24) = 3.234; *p* = 0.0035; Cohen’s dz = 0.647). Normalized contrast was also significantly higher under NBI than under white light (0.322 ± 0.178 vs. 0.147 ± 0.106; mean paired increase, 0.1745; 95% CI, 0.1120–0.2369; t(24) = 5.767; *p* < 0.0001; Cohen’s dz = 1.153). Wilcoxon signed-rank sensitivity analyses were concordant for absolute contrast (V = 266; *p* = 0.0054) and normalized contrast (V = 313; *p* < 0.0001). Shapiro–Wilk p values for the paired absolute- and normalized-contrast differences were 0.975 and 0.187, respectively.

The direction of the paired difference was also examined. Under white light, tumor tissue appeared brighter than adjacent normal tissue (a reversed direction relative to the expected pattern) in 4 of 25 patients; under NBI, this reversed direction was observed in 2 of 25 patients (Case 2, uterine sarcoma; Case 7, colorectal adenocarcinoma). The signed tumor-to-normal difference (normal minus tumor) was significantly larger under NBI than under white light (67.060 ± 49.206 vs. 40.739 ± 34.536; t(24) = 2.965; *p* = 0.0067), consistent with a significant tissue-type-by-modality interaction (interaction contrast, −26.321 ± 44.380; t(24) = −2.965; *p* = 0.0067). At the individual-patient level, absolute contrast increased under NBI relative to white light in 19 of 25 patients (76%) and decreased in 6 of 25 patients (24%: Cases 1, 10, 14, 16, 18, and 23); the two patients with cutaneous melanoma metastases (Cases 1 and 23) were both among these six, a hypothesis-generating pattern potentially reflecting attenuation of hemoglobin-dependent NBI contrast by melanin pigmentation. Group sizes by primary tumor origin were too small for formal subgroup testing and are summarized descriptively in Table S1.

## 4. Discussion

### 4.1. Principal Findings

This exploratory study links intraoperative optical appearance to both tissue pathology and digital image contrast, building on the preliminary histological observations summarized in Section 3.3. Across all 25 patients, both white light and NBI distinguished tumor from adjacent normal tissue, but NBI produced significantly greater absolute tumor-to-normal contrast. The mean absolute contrast increased from 44.287 under white light to 69.628 under NBI, representing a mean paired increase of 25.342 units (95% CI, 9.169–41.514; *p* = 0.0035) and a moderate-to-large within-patient effect (Cohen’s dz = 0.647). Normalized contrast analysis and Wilcoxon sensitivity analysis supported the same conclusion. It is important to state explicitly what this study does and does not demonstrate. These data show that, for regions of tissue visually identified as tumor or normal brain, NBI produced a greater quantitative gray-value difference than white light in this retrospective cohort. These data do not demonstrate improved diagnostic accuracy for tumor identification, more accurate delineation of microscopic tumor infiltration, a more complete surgical margin, reduced local recurrence, or improved survival. The biological mechanisms proposed below (Section 4.2) are hypotheses consistent with the known optical basis of NBI and were not directly measured in this study.

### 4.2. Biological and Optical Interpretation

NBI contrast is generated primarily by wavelength-selective hemoglobin absorption rather than intracellular accumulation of a fluorophore [13,14]. Although not directly measured in this study, a grayish or brown-gray tumor appearance is hypothesized to reflect altered microvascular density, vessel caliber, oxygenation, tissue scattering, or their combination; these mechanisms remain hypotheses rather than explanations established by our data. The correspondence with tumor cells does not establish that NBI directly visualizes cells, nor that a larger measured gray-value difference reflects better detection of microscopic tumor infiltration or a more accurate surgical margin: it demonstrates only that visually identified tumor and normal regions differed more under NBI than white light in this dataset. Notably, in Patient No. 14—one of the three patients with histopathologically confirmed tumor in the grayish-appearing region (Figure 2)—the quantitative absolute gray-value contrast was nonetheless lower under NBI than under white light (Section 3.4), illustrating that qualitative optical appearance and pixel-level quantitative contrast may diverge in individual patients. NBI likely depicts a vascular phenotype spatially associated with tumors rather than tumor cells themselves; this distinction is important when interpreting false-positive signals from blood, reactive tissue, or non-neoplastic vascular changes.

The susceptibility to blood is mechanistically expected: the same hemoglobin absorption that enhances vessels can cause darkening when blood covers the field. Standardized hemostasis, irrigation, camera geometry, illumination, exposure control, and frame-selection criteria are therefore prerequisites for reproducible quantitative work. A normalized contrast metric and within-field pairing can reduce, but not eliminate, the effects of global illumination and device processing.

### 4.3. Relationship with 5-ALA and Multimodal Visualization

5-ALA is currently the only intraoperative optical adjunct with randomized evidence in malignant glioma [7,8] and is therefore the most established benchmark against which a new intraoperative optical technique is naturally discussed in this field; however, this study did not directly compare NBI with 5-ALA in the same patients. NBI offers a different, agent-free source of contrast that could complement rather than replace 5-ALA, indocyanine green videoangiography, neuronavigation, intraoperative ultrasound, or neurophysiological mapping [26,27,28], using the exoscope as a practical multimodal platform [20,21,22,23].

### 4.4. Relevance to the Brain Metastasis Margin

Histological studies have challenged the assumption that every brain metastasis is sharply separated from surrounding brain. Microscopic invasion varies by primary tumor and growth pattern and may contribute to local failure [3,4,5,6]. Selected series suggest that microsurgical or supramarginal resection can improve local control, although patient selection and neurological safety remain critical and high-level evidence is limited [29,30,31]. An optical technique that helps identify suspicious tissue could assist margin-directed sampling or resection, but any extension beyond the visible lesion must remain subordinate to functional anatomy and established oncological treatment planning.

### 4.5. Quantitative Image Analysis and Future Validation

ImageJ and Fiji are established open-source platforms for reproducible biomedical image measurement [24,25]. In the present study, the patient-level paired design avoided treating pixels or ROIs as independent observations. Absolute tumor-to-normal contrast was significantly higher with NBI than with white light, and the normalized contrast increased from 0.147 ± 0.106 to 0.322 ± 0.178 (mean paired increase, 0.1745; 95% CI, 0.1120–0.2369; *p* < 0.0001; Cohen’s dz = 1.153). Future studies should prospectively lock camera settings, ROI rules, contrast formulas, exclusion criteria, and histopathological registration. Blinded multi-reader assessment, inter-rater reliability, receiver-operating-characteristic analysis, and external validation will be needed before defining an intraoperative decision threshold.

### 4.6. Clinical Implications

NBI is rapidly repeatable and entails no drug exposure. Its most plausible immediate role is as an adjunct used after hemostasis to prompt closer inspection, targeted sampling, or comparison with other modalities. In eloquent regions, NBI cannot justify resection of tissue that would otherwise be functionally unsafe. In non-eloquent regions, prospective trials would be required to determine whether NBI-guided margin management improves extent of resection or local control without increasing neurological morbidity.

## 5. Limitations

This study is retrospective, single-center, exploratory, and based on 25 patients. Although the complete paired gray-value dataset was analyzed, the cohort remains modest and no external validation was performed. All operations and NBI classifications were performed by a single surgeon, which provided internal consistency but means that inter-observer reliability could not be assessed; neuropathologists were not blinded to the intraoperative NBI designation, and blinding of the image analysts to histopathological results was not documented for the quantitative cohort; tumor and normal-tissue ROIs in the 22 patients without paired histology were selected from the surgeon’s visual impression rather than independent confirmation (Section 2.6); paired negative-margin sampling was limited by safety; and spatial registration between images and specimens may be imperfect. No a priori sample-size calculation was performed, and exposure, gain, and white balance were adjusted automatically by the exoscope system rather than held constant between modalities. The exact anatomical distance between paired sampling sites was not prospectively recorded; therefore, the present histopathological observations cannot establish the spatial accuracy of the apparent NBI margin, and a future prospective study should use predefined sampling distances and intraoperative image-to-specimen registration. Image appearance may be affected by bleeding, irrigation, camera angle, focus, zoom, exposure, white balance, compression, monitor processing, and retrospective ROI selection. The histopathological comparison was available only in three selectively sampled patients (six specimens), providing preliminary observations rather than diagnostic validation, so sensitivity and specificity cannot be estimated. At the individual-patient level, NBI increased absolute contrast in 19 of 25 patients but decreased it in 6 of 25 patients, indicating that the benefit was not uniform and may vary by tissue or pigmentation characteristics (Section 3.4). Finally, this study did not evaluate whether NBI guidance improves extent of resection, neurological outcome, local recurrence, or survival.

## 6. Conclusions

In this retrospective, exploratory, single-center study, the grayish NBI appearance corresponded to metastatic tumor in three selectively sampled, spatially registered specimen pairs, and NBI significantly increased absolute and normalized tumor-to-normal gray-value contrast relative to white light across the full 25-patient cohort, although this increase was not observed in every patient. These findings support NBI as a feasible, agent-free adjunct that warrants prospective, blinded validation before any claim of diagnostic accuracy or clinical benefit can be made.

## Figures and Tables

**Table 1:** Clinicopathological characteristics and intraoperative gray-value contrast of the 25 patients.

Case	Age, Years	Sex	Primary Site	Histology	Side	Location	Absolute Contrast, WL/NBI	Normalized Contrast, WL/NBI
1	78	M	Skin	Malignant melanoma	Left	Parietal lobe	125.2/68.9	0.442/0.518
2	58	F	Uterus	Sarcoma	Left	Insular region	10.3/21.6	0.028/0.151
3	56	M	Colon	Adenocarcinoma	Right	Temporal lobe	66.9/118.2	0.171/0.393
4	56	M	Liver	Adenocarcinoma	Right	Occipital lobe	48.0/50.6	0.148/0.533
5	82	M	Lung	Adenocarcinoma	Left	Frontal lobe	28.0/62.7	0.085/0.220
6	77	F	Colon	Adenocarcinoma	Right	Temporal lobe	49.5/106.8	0.135/0.410
7	64	M	Colon	Adenocarcinoma	Right	Frontal lobe	6.5/10.5	0.020/0.076
8	64	M	Bladder	Transitional cell carcinoma	Left	Parietal lobe	20.3/93.2	0.060/0.432
9	78	M	Colon	Adenocarcinoma	Right	Parietal lobe	70.7/110.9	0.210/0.571
10	69	F	Lung	Adenocarcinoma	Right	Frontal lobe	13.5/2.5	0.038/0.007
11	73	M	Esophagus	Squamous cell carcinoma	Left	Frontal lobe	50.9/59.3	0.138/0.234
12	80	M	Ureter	Neuroendocrine carcinoma	Left	Parietal lobe	7.8/27.0	0.024/0.123
13	80	F	Breast	Adenocarcinoma	Right	Cerebellum	25.4/101.5	0.075/0.525
14	77	M	Thyroid	Anaplastic carcinoma	Left	Occipital lobe	92.2/64.8	0.383/0.442
15	64	M	Esophagus	Adenocarcinoma	Left	Frontal lobe	34.5/66.1	0.139/0.308
16	59	F	Lung	Large cell carcinoma	Midline	Cerebellum	37.9/34.4	0.163/0.302
17	62	F	Lung	Adenocarcinoma	Right	Parietal lobe	18.5/34.9	0.076/0.191
18	82	M	Thyroid	Anaplastic carcinoma	Right	Parietal lobe	45.4/22.2	0.178/0.094
19	72	M	Colon	Adenocarcinoma	Left	Cerebellum	27.2/45.5	0.101/0.331
20	74	M	Lung	Adenocarcinoma	Left	Frontal lobe	77.7/103.1	0.247/0.321
21	68	M	Colon	Adenocarcinoma	Right	Frontal lobe	66.3/167.8	0.204/0.618
22	65	M	Bladder	Transitional cell carcinoma	Left	Parietal lobe	66.1/165.1	0.180/0.559
23	50	F	Skin	Malignant melanoma	Left	Insular region	41.5/24.0	0.145/0.073
24	67	F	Thyroid	Anaplastic carcinoma	Left	Frontal lobe	67.2/117.9	0.261/0.386
25	58	M	Colon	Adenocarcinoma	Right	Temporal lobe	9.7/61.1	0.029/0.225

Abbreviations: F, female; M, male; WL, white light; NBI, narrow band imaging; GV, gray value. Absolute contrast is defined as |GV(tumor) − GV(normal)| and normalized contrast as absolute contrast divided by the mean background GV, both in 8-bit gray-value units (0–255), as described in Section 2.6; values are shown as white light/NBI.

## Data Availability

The de-identified data presented in this study are available from the corresponding author upon reasonable request.

## Supplementary Materials

The following supporting information can be downloaded at: https://www.mdpi.com/article/10.3390/cancers18193171/s1, Figure S1: Patient flow diagram; Table S1: Descriptive summary of absolute tumor-to-normal gray-value contrast (white light vs. NBI) by primary tumor origin for the 25 patients in this study.
