# Supplementary material for: Histopathological and Quantitative Correlates of Intraoperative Narrow Band Imaging in Brain Metastasis Surgery
Source: Cancers (Basel). 2026 Oct 1;18(19):3171. doi: 10.3390/cancers18193171 (PMC13664459; doi:10.3390/cancers18193171)
Supplement: Supplementary file 1 [file cancers-18-03171-s001.zip › cancers-4581565-supplementary.pdf]

Supplementary Materials

Patients undergoing surgical resection of brain metastasis, Nippon Medical School Hospital, June 2022 – June 2025 (n ≈ 35)

↓

Excluded (n = 10):

- Dedicated NBI imaging not performed or recorded during the operation, or
- Same patient already represented in the cohort (avoided duplicate counting)

↓

Patients with complete paired white-light and NBI images included in the quantitative gray-value contrast analysis (n = 25)

↓

Subset in whom paired histological sampling of a grayish-appearing region and an adjacent whitish-appearing region was performed (n = 3)

Figure S1. Patient flow diagram. This flow diagram is referenced in Section 2.1 (Patient Population) of the main text.

Table S1. Descriptive summary of absolute tumor-to-normal gray-value contrast (white light vs. NBI) by primary tumor origin for the 25 patients in this study.

Primary tumor origin	n	Mean absolute contrast, white light	Mean absolute contrast, NBI	Mean change (NBI − white light)
Colorectal	7	49.6	96.9	+47.3
Lung	5	25.0	36.0	+11.0
Thyroid	3	68.3	68.3	0.0
Skin (melanoma)	2	83.3	46.5	−36.9
Bladder	2	43.2	129.1	+85.9
Esophageal	2	42.7	62.7	+20.0
Uterine (sarcoma)[a]	1	10.3	21.6	+11.3
Hepatic[a]	1	48.0	50.6	+2.6
Ureteral[a]	1	7.8	27.0	+19.2
Breast[a]	1	25.4	101.5	+76.0

[a] n = 1; the value shown is that single patient's observation rather than a group mean. Absolute contrast is defined as |gray value(tumor) − gray value(normal)|, in 8-bit gray-value units (range of 0–255), as described in Section 2.6 of the main text. Group sizes were small and are presented descriptively only; no formal between-group statistical testing was performed. This table is referenced in Section 3.4 of the main text.
